# Novel *COL4A1* mutations identified in infants with congenital hemolytic anemia in association with brain malformations

**DOI:** 10.1038/s41439-020-00130-w

**Published:** 2020-11-27

**Authors:** Hiromi Ogura, Shouichi Ohga, Takako Aoki, Taiju Utsugisawa, Hidehiro Takahashi, Asayuki Iwai, Kenichiro Watanabe, Yusuke Okuno, Kenichi Yoshida, Seishi Ogawa, Satoru Miyano, Seiji Kojima, Toshiyuki Yamamoto, Keiko Yamamoto-Shimojima, Hitoshi Kanno

**Affiliations:** 1 Department of Transfusion Medicine and Cell Processing, Tokyo Women’s Medical University, Tokyo, Japan; 2 Department of Pediatrics, Graduate School of Medical Sciences, Kyushu University, Fukuoka, Japan; 3 Department of Neonatology, Musashino Red Cross Hospital, Tokyo, Japan; 4 Department of Pediatrics, Shikoku Central Hospital, Ehime, Japan; 5 Department of Hematology and Oncology, Shizuoka Children’s Hospital, Shizuoka, Japan; 6 Center of Advanced Medicine and Clinical Research, Nagoya University, Aichi, Japan; 7 Department of Pathology and Tumor Biology, Kyoto University Graduate School of Medicine Faculty of Medicine, Kyoto, Japan; 8 Institute for the Advanced Study of Human Biology (WPI-ASHBi), Kyoto, Japan; 9 Department of Medicine, Center for Hematology and Regenerative Medicine, Karolinska Institute, Stockholm, Sweden; 10 M&D Data Science Center, Tokyo Medical and Dental University, Tokyo, Japan; 11 Department of Pediatrics, Nagoya University Graduate School of Medicine, Nagoya, Japan; 12 Institute of Medical Genetics, Tokyo Women’s Medical University, Tokyo, Japan

**Keywords:** Genetic testing, Disease genetics

## Abstract

Genetic causes of undiagnosed hemolytic anemia in nineteen patients were analyzed by whole-exome sequencing, and novel *COL4A1* variants were identified in four patients (21%). All patients were complicated with congenital malformations of the brain, such as porencephaly or schizencephaly. In these patients, hemolysis became less severe within 2 months after birth, and red cell transfusion was no longer required after 50 days, whereas chronic hemolysis continued.

Congenital hemolytic anemia is caused by genetic abnormalities of the red cell membrane, enzyme, or hemoglobin. We have been performing research studies on congenital hemolytic anemia. As a result, extensive analyses of hemoglobin, red cell membrane, and enzymes have been performed. However, approximately 40% of patients remained undiagnosed (personal data). Recently, we encountered infantile patients with hemolytic anemia in whom novel *COL4A1* variants were identified. Here, detailed information on the patients and the identified variants is reported.

This study was performed in accordance with the Declaration of Helsinki and was approved by the Ethics Committee of the institution. After receiving written an informed consent from the patients’ families, we obtained blood samples from patients and their parents. Genomic DNA was extracted using a standard protocol. Using trio samples, whole-exome sequencing (WES) was performed, as described previously^1^. The extracted data were mapped to a reference genome (GRCh37/hg19), annotated, and filtered in accordance with the method described previously^1^. The possible candidate variants were reconfirmed by Sanger sequencing.

Nineteen infants with undiagnosed congenital hemolytic anemia were included in this study. Immunological hemolysis, unstable hemoglobinopathies, red cell membrane defects and red cell enzymopathies were ruled out by routine laboratory tests, including direct a antiglobulin test, isopropanol test, osmotic fragility test, eosin 5′-maleimide binding test, acidified glycerol lysis time, and reduced glutathione and 15 red blood cell enzyme activities.

Through WES, four novel *COL4A1* missense variants were identified among 19 patients with undiagnosed infantile hemolytic anemia (4/19 = 21%): NM_001845.6:c.2354G>T [p.Gly785Val], c.2537G>A [p.Gly846Glu], c.2788G>A [p.Gly930Ser] and c.2843G>A [p.Gly948Asp] (Table 1). In the WES data, there were no other possible candidate variants related to hemolytic anemia. The identified *COL4A1* variants were reconfirmed by Sanger sequencing. According to the ACMG recommendation, all variants were evaluated as “pathogenic” or “likely pathogenic”^2^.

**Table 1:** Results of this study.

	Case 1	Case 2	Case 3	Case 4
Mutation	chr13:110831374	chr13:110830500	chr13:110829313	chr13:110829258
	NM_001845.6:c.2354G>T	NM_001845.6:c.2537G>A	NM_001845.6:c.2788G>A	NM_001845.6:c.2843G>A
	p.Gly785Val	p.Gly846Glu	p.Gly930Ser	p.Gly948Asp
CADD_phred	23.1	24.1	35	24.3
ACMG criteria	PS1, PS2, PM2, PP3, and PP4	PS2, PM2, PP3, and PP4	PS2, PM2, PP3, and PP4	PS2, PM2, PM5, PP3, and PP4
Interpretation	Pathogenic	Likely pathogenic	Likely pathogenic	Pathogenic
Inheritance	de novo	de novo	de novo	de novo
Gender	M	F	F	F
Age (month)	0	2	2	3
Family history	–	–	–	–
Period of gestation (week)	39	37	35	37
Birth weight (g)	2190	2467	1624	2336
Test day (day)	0	47	81	115
Hb (g/dL)	9.3	6.8	6.7	7.4
MCV (fL)	127	99	110	90
MCHC (%)	31.4	31.9	29.9	32.7
Retics (%)	16.4	9.2	14	ND
LDH (U/L)	582	302	242	847
Hp (mg/dL)	1	<10	<7.1	<10
Osmotic fragility	ND	ND	ND	−
EMA (% of Ct)	ND	87.6	98.1	94.2
RBC morphology	Normal	Poikilocytes	Shizocytes	Anisocytes
Icterus gravis neonatorum	+	–	+	+
Last blood transfusion date (day)	6	50	45	51
Splenomegaly	–	–	–	–
Phototherapy	+	–	+	+
Exchange blood transfusion	7 times	–	–	–
Improvement of anemia	+	+	+	+
Others	Microcephaly, paraventricular calcification, cataract	Schizencephaly, cataract, macroglossia	Microcephaly, porencephaly with paraventricular carcification	Hydrocephaly, epidermolysis bullosa

EMA eosin 5′-maleimide, ND not detected

Clinical information on the patients is also summarized in Table 1. All patients were born with low birth weight in association with moderate to severe neonatal hemolytic anemia. The prenatal medical histories of all patients were not remarkable. Morphological changes in the peripheral blood smear were observed in three patients (Cases 2, 3, and 4). All patients showed spontaneous remission of hemolytic anemia within 2 months after birth and no longer required red cell transfusion after 50 days. All patients were complicated with congenital anomalies in the central nervous system, such as porencephaly or schizencephaly.

In 2005, *COL4A1* was identified as a causative gene for hereditary porencephaly^3^. Variants in *COL4A1* have been shown to be associated with a broad range of disorders, including small-vessel brain disease of various severities, including polencephaly, variably associated with eye defects (retinal arterial tortuosity, Axenfeld-Rieger anomaly, and cataract) and systemic findings (kidney involvement, muscle crumps, cerebral aneurysms, Raynaud phenomenon, cardiac arrhythmia, and hemolytic anemia)^4^.

To date, seven patients with a *COL4A1*-related disorder in association with hemolytic anemia have been reported. In 2013, Yoneda et al. reported that five among fifteen patients with *COL4A1*-related disorder had hemolytic anemia^5^, indicating that hemolytic anemia may be one of the related features of *COL4A1*-related disorder. Interestingly, five patients showed improvement of anemia, with the longest at 6 months of age. In 2016, Tomotaki et al. reported a male infant with *COL4A1*-related disorder with severe hemolytic jaundice. His anemia improved after the red cell transfusion on day 29 without recurrence^6^. Maisonneuve et al. reported a fetal case with severe anemia in association with cerebral ischemohemorrhagic damage revealed by ultrasonography, in whom a de novo *COL4A1* mutation was identified^7^.

The clinical and hematological characteristics identified in this study are similar to those in previous reports^5,6^. The present four patients had severe jaundice and/or progressive anemia from birth, although there was no family history of anemia and jaundice. Three patients (Cases 2, 3, and 4) had icterus gravis neonatum and received phototherapy. Case 1 received blood transfusion seven times. These hematological features were almost certainly derived from *COL4A1* mutations.

*COL4A1* encodes COL4A1 (α1 chain), which consists of an amino-terminal 7S domain, a triple-helix forming collagenous domain, and a carboxyl-terminal noncollagenous domain. The triple-helix forming collagenous domain consists of glycine-X-Y amino acid repeats, which are essential for the formation of type IV collagen protein^8^. COL4A1 forms a triple-helical structure with COL4A2 (α2 chain) by combining as heterotrimers with a 2:1 stoichiometry (alpha1-alpha1-alpha2), forming the sheet-like network^9^.

The four *COL4A1* variants identified in this study result in the substitutions of glycine for another amino acid residue in one of the glycine-X-Y repeats. The amino acid position of the mutation identified in Case 4, p.Gly948Asp, was the same as Case 4 reported by Yoneda et al., p.Gly948Ser, who did not show hemolytic anemia. The same amino acid of p.G785V identified in Case 1 was also affected in the case previously reported (p.G785E), with intrauterine stroke and anterior segment dysgenesis^10^.

The mechanisms of hemolytic anemia in patients with *COL4A1*-related disorder have not yet been established. However, some proposed explanations have been considered. One explanation is that dysfunction of basement membranes leads to red cell destruction through the vasculature or reticuloendothelial system and effective transmigration of red cells or erythroid progenitor cells. Janowska-Wieczorek et al. reported that peripheral blood CD34+ cells strongly express collagen type IV degrading gelatinases, matrix metalloproteinases-2 (MMP-2) and MMP-9^11^. They suggested that type IV collagens expressed in bone marrow sinusoidal basement membranes influence the transmigration of blood progenitor cells. Infantile hemolytic anemia observed in four patients with *COL4A1* variants spontaneously improved later. Morphological findings of the peripheral blood smear, such as schiozocytes and poikilocytes, were observed in peripheral blood smears in two patients. Transient hemolytic anemia and abnormal erythrocyte morphology resemble the findings of hereditary elliptocytosis during infancy. Transient hemolysis may depend on the development and growth of skeletal and nonskeletal red cell components.

It has been reported that free 2,3-diphosphoglycerate (DPG), presented in neonatal RBCs as a consequence of diminished binding to fetal Hb, may render HE susceptible to in vivo fragmentation^12^. The developmental switch from fetal to adult hemoglobin, by diminishing available free 2,3-DPG, may explain the abatement of hemolytic anemia that accompanies maturation.

In conclusion, *COL4A1*-related disorder should be listed as one of the differential diagnoses when we encounter infants with undiagnosed hemolytic anemia and congenital malformations of the brain. We consider that permanent management for hemolytic anemia is not necessary for patients with *COL4A1*-related disorders. Further studies are required to clarify how *COL4A1* mutations are involved in anemia.

## Data Availability

The relevant data from this Data Report are hosted at the Human Genome Variation Database at 10.6084/m9.figshare.hgv.2930, 10.6084/m9.figshare.hgv.2933, 10.6084/m9.figshare.hgv.2936, 10.6084/m9.figshare.hgv.2939.
